# Public private partnership in vascular surgery

**DOI:** 10.1590/S1679-45082014GS3029

**Published:** 2014

**Authors:** Cynthia de Almeida Mendes, Alexandre de Arruda Martins, Marcelo Passos Teivelis, Sérgio Kuzniec, Nelson Wolosker

**Affiliations:** 1 Hospital Israelita Albert Einstein, São Paulo, SP, Brazil.

**Keywords:** Vascular surgery procedures/economics, Health management, Unified Health System, Angioplasty/economics, Aortic aneurysm

## Abstract

**Objective:**

To describe and analyze the results of a public-private partnership between the Ministry of Health and a private hospital in a project of assistance and scientific research in the field of endovascular surgery. Methods: The flows, costs and clinical outcomes of patients treated in a the public-private partnership between April 2012 and July 2013 were analyzed. All patients underwent surgery and stayed at least one day at the intensive care unit of the private hospital. They also participated in a research protocol to compare two intravenous contrast media used in endovascular surgery (iodinated contrast and carbon dioxide).

**Results:**

A total of 62 endovascular procedures were performed in 57 patients from the public healthcare system. Hospital and endovascular supplies expenses were significantly higher as compared to the amount paid by the Unified Health System (SUS - Sistema Único de Saúde) in two out of three disease groups studied. Among outpatients, the average interval between appointment and surgery was 15 days and, in hospitalized patients 7 days. All procedures were successful with no conversion to open surgery. The new contrast medium studied - carbon dioxide – was effective and cheaper.

**Conclusion:**

The waiting time for patients between indication and accomplishment of surgery was significantly reduced. Public-private partnerships can speed up care of patients from public health services, and generate and improve scientific knowledge.

## INTRODUCTION

Arterial diseases are severe conditions that most frequently affect people between the 6th and 8th decade of life.^(1)^ Improvement of public health services led to increased life expectancy of the population;^(2)^ thus, arterial diseases are more prevalent and generate high demand to the Unified Health System (SUS - Sistema Único de Saúde).

Treatment of these diseases is complex and frequently requires specialized surgery in tertiary care centers. These surgical procedures require sophisticated equipment, highly trained teams and intensive care unit (ICU) equipped for postoperative care.

Despite considerable advances at the SUS, a large number of patients face difficulties when seeking this type of treatment, especially because of the insufficient number of hospital inpatient vacancy and limited access to high-complexity hospitals.

The high demand of patients at the public health system and the limited resources lead to poorer prognosis of these patients. Silvany Neto et al. demonstrated that in patients with occlusive peripheral artery disease (PAD), delay in carrying out exams leads to higher amputation rates.^(3)^ The delay in treating inpatients has a negative impact on the outcome and increases mortality.^(4)^ Turnbull et al. and Oram et al. noticed that a delay in treating patients with abdominal aorta aneurysm (AAA) increases the incidence of ruptures and results in higher mortality rate.^(5, 6)^

In order to improve care and prognosis of these patients, the structural deficiencies of the SUS could be minimized, in part, by public-private partnerships (PPP) between the government and duly equipped private hospitals, which would provide the complete infrastructure needed for these procedures and meet this demand from the public health system.

In 2009, the *Hospital Israelita Albert Einstein* (HIAE) through the Program of Institutional Development of SUS (PROADI-SUS) established a PPP with the Ministry of Health to perform endovascular procedures in SUS patients suffering from femoropopliteal and aortoiliac obstructive disease, and abdominal aorta aneurysms.

The aim of the PPP was the treatment of patients with femoropopliteal and aortoiliac obstructive disease and AAA, providing the structure of HIAE for surgical procedures and post-operative recovery, based on a health research for evaluation of a new intra-arterial contrast, carbon dioxide (CO2), for carrying out such endovascular procedures.

## OBJECTIVE

The objective of this article is to describe and analyze the results of a pioneer public-private partnership between the Ministry of Health and a private hospital in a project of assistance and scientific research in the field of endovascular surgery, which can serve as a basis for planning future PPPs.

## METHODS

Between April 2012 and July 2013, the PPP provided treatment at the HIAE for 48 patients suffering from DAP in femoropopliteal or aortoiliac arteries, and 9 patients presenting AAA. A total of 62 endovascular procedures were performed.

The patients undergoing treatment participated in a health research project authorized by the Research Ethics Committee (CAAE: 00794012.5.0000.0071) of the hospital. All patients filled out and signed the Informed Consent Form, which was one inclusion criterion of the study.

The majority of the patients were referred by Specialty Outpatient Clinics (AMES - Ambulatórios Médicos de Especialidades) of the neighborhoods of Capão Redondo, Jardim São Luís and Jardim Ibirapuera, in the Southern region of the city of Sao Paulo (SP), which represent the area covered by Municipal Hospital of M’boi Mirim − Dr. Moysés Deutsch (HMMD). This hospital was the partner of HIAE in this PPP. The inpatients waiting for endovascular procedures in other hospitals of the region were also included in the project.

Prior to starting the project, medical managers of the healthcare units in the region disseminated information and orientations, targeting expert physicians at the units in order to establish a referral flow of eligible cases.

Patients seen at the healthcare units during routine vascular surgery examination who suffered from the aforementioned diseases and for whom surgery was indicated, were referred to the outpatient screening clinic related to this project, located at the HMMD. Preoperative tests were requested by the outpatient screening clinic in the HMMD or by the Primary care Units (UBS - Unidade Básica de Saúde), both part of the SUS services. Preoperatorive angiotomography of the patients was performed at the Radiology Service of the HMMD.

The physicians participating in the project, who work at the HMMD and HIAE, were in charge of screening procedures at the outpatient clinics.

All screened cases were discussed by the team in charge of the project at weekly meetings at the HIAE, when the physicians analyzed whether inclusion criteria had been met. Patients who had been referred to the outpatient clinic but failed to meet the inclusion criteria of the study were referred back to follow the routine flow of SUS.

After enrolling patients in the project, surgery was scheduled at HIAE. On the day of surgery patients were admitted to the hospital and were directly referred to the endovascular operating room, where all procedures were performed. The endovascular room is fully equipped with the necessary fluoroscopy and anesthesia equipment. The quality of equipment allowed surgeons to safely carry out the procedures and perform the study comparing the new intravascular contrast (CO_2_) to the conventional iodinated contrast.^(7)^

After the procedure, the patients were systematically sent to the ICU HIAE, where they remained until the day after the procedure, when, then, if there were no clinical contraindication, were transferred to HMMD to another hospital of origin or even released to their homes.

Intrahospital costs had been previously estimated, since all patients - regardless of their clinical *status* after surgery - spent 24 hours at the ICU of HIAE.

All endovascular supplies (catheters, guidewires, stents, balloons, endografts) and contrast media used in the procedures were recorded for cost analyses.

Transfer of patients to the hospital of origin was done using an ICU-ambulance assigned for this project.

After hospital discharge, patients treated by this project continued postoperative follow-up at the services of origin and at the outpatient clinic of HMMD.

Data was adquired in real time, and consisted of weekly updates of the spreadsheet containing medical data, costs, and clinical progression of the patients.

All costs regarding endovascular material and hospital stay were registered for later analysis and comparison.

The demographic characteristics of the study population, referral flow, waiting time for the procedure, hospital costs and cost of endovascular supplies were analyzed.

## RESULTS

From April 2012 to August 2013, a total of 62 procedures were performed in 57 patients referred from the SUS. There were 44 femoropopliteal angioplasties, 10 infrarenal AAA repairs, and 8 aortoiliac angioplasties.

Forty-four patients were referred by the primary care unit (UBS) of the region covered by the HMMD, and 14 patients had been admitted to other hospitals in Sao Paulo and were waiting for endovascular surgery.

The mean time between the outpatient screening clinic first visit and the surgical procedure was 15 days, range 5-25 days. All exams and preoperative assessments were performed during this period. In the case of inpatients, the maximum surgical waiting time was 7 days.

The study population consisted of 37 men and 20 women. The mean age was 63.56 years (range 37-82 years). Out of these patients 46 (80.7%) suffered from hypertension, 26 (45.6%) from diabetes, and 3 (5.2%) from congestive heart failure. Hypercholesterolemia was observed in 25 (43.8%), coronary insufficiency in 8 (14%), and 37 (64.9%) were smokers.

Trophic lesions associated with ischemic pain at rest were observed in 42 PAD patients (37 femoropopliteal disease and 5 with aortoiliac involvement). Intermittent claudication was present in three patients of the femoropopliteal group and in two patients of the aortoiliac group. Ischemic pain at rest without trophic lesion was present in one patient of the aortoiliac group.

Only four patients stayed at the ICU-HIAE for more than 1 day (one patient for 2 more days, and three for 1 more day). The mean length of stay was 1.1 day at the HIAE.

The 30-day mortality in the study group was 4 cases (12%), all were patients with femoropopliteal PAD who developed cardiac complications postoperatively.

Hospital expenses are shown in table 1.

**Table 1:** Hospital expenses according to procedure

Procedure	Quantity	Minimum hospital expense (R$)	Maximum hospital expense (R$)	Mean hospital expense (R$)
FPA	44	4,780.44	34,333.42	8,834.36
AIA	8	2,769.46	10,737.0	6,218.00
EVAR	10	6,207.27	15,400.89	9,258.78

FPA: femoropopliteal angioplasties; AIA: aortoiliac angioplasties; EVAR: endovascular aortic aneurysm repair; R$: Reals.

The expenses were higher in the group treated for aortic aneurysm.

Table 2 presents the expenses related to with endovascular supplies.

**Table 2:** Expenses with endovascular supplies by procedure

Procedure	Quantity	Minimum cost of supplies (R$)	Maximum cost of supplies (R$)	Mean cost of supplies (R$)
FPA	44	1,664.64	23,452.00	7,413.25
AIA	8	1,782.00	8,052.00	4,672.75
EVAR	10	17,372.00	41,816.67	31,727.73

FPA: femoropopliteal angioplasties; AIA: aortoiliac angioplasties; EVAR: endovascular aortic aneurysm repair; R$: Reals.

The expenses with endovascular supplies in AAA were four-fold higher than lower limb angioplasties and seven-fold higher than iliac angioplasties.

Hospital and endovascular supplies costs were compared to reimbursement values of SUS and are displayed in table 3.

**Table 3:** Hospital and endovascular supplies costs at Hospital Israelita Albert Einstein (HIAE) and reimbursement values paid by the Unified Health System (SUS)

Group	Type of expenditure	n	Expenses HIAE (R$)	Reimbursement SUS (R$)	p value
			Mean (SD)			
Aneurysms	Hospitalar	10	9.258,80 (3183,20)	1.541,30	<0,001
	Supplies	10	31.727,70 (7754,70)	25.455,00	0,031
Aortoiliac	Hospitalar	8	6.218,00 (2.223,60)	1.541,30	0,001
	Material	8	4.672,70 (1.998,30)	5.340,00	0,376
Femoropopliteal	Hospitalar	44	8.834,40 (5.040,80)	999,00	<0,001
	Material	44	7.413,20 (4.444,00)	4.965,00	0,001

SD: Standard deviation. R$: Reals.

Cost at HIAE was significantly higher than the reimbursement of SUS, with exception of the cost of material in the aortoiliac group.

## DISCUSSION

Symptomatic occlusive PAD and AAA greater than 5cm are increasingly more frequent diseases and require surgical treatment. The number of patients waiting for surgery in Brazil is greater than the number of hospital beds available at the SUS, and treatment delay may increase the rate of amputations and deaths.^(3, 6)^

The patient referral flow established in this PPP project allowed the treatment of 57 patients who were waiting for surgery in the Southern region of the city of Sao Paulo. The integration of information between the UBS and the outpatient clinic of the project was essential to provide patients with fast access to the treatment they needed.

Patients who seek the public health sector frequently have limited access to services that provide treatment for these diseases. Furthermore, when patients are finally admitted to these services, the disease is already in an advanced stage and delay in providing effective treatment contributes to poorer prognosis. Nunes et al. showed that 96.2% of inpatients with occlusive PAD at a tertiary service already presented trophic lesions; in 62.3% of them the extension of the lesions hindered further attempts of revascularization prior to amputation.^(8)^

One of the main hurdles to the universal access to care at the SUS is access to a specialist, such as a vascular surgeon. A partnership with private health systems in PPP can, as seen in this group of patients, minimize this problem.^(9)^

Patients treated under this project came from hospitals and UBS from the SUS, and represent a real sample of the population assisted by the public health system, as well as the costs of these diseases for the public health system. These patients are often seriously ill, with many comorbidities and high surgical risk. In this project, the mortality was 12% - all patients in the femoropopliteal group, and was as expected by the literature.^(10)^

The technical success rate of the endovascular procedures carried out by our group during this project was 100%. All surgeries finished successfully with no convertion to open surgery. Limb salvage rate among patients suffering from PAD was 92%; four limbs were amputated and no surgical complication was reported in the AAA group.

The surgery waiting time during this project was the interval needed to perform complementary preoperative exams. The entire preoperative assessment was conducted at the SUS through the project’s outpatient clinic at the HMMD. We consider that the waiting time in this PPP (mean of 15 days) was considerably short. There is scarce recent literature on waiting time for arterial surgeries. A previous Brazilian study reported a mean waiting time for preoperative arteriography (which was replaced by angiotomography, in this study) was 16.3 days, and inpatients had to wait, in average, 32 days to undergo a revascularization procedure.^(8)^ There are no Brazilian studies on how long outpatients have to wait to perform these procedures. Nevertheless, in public health services of the city of Sao Paulo (where this population would receive medical care), the waiting period could be longer than the previously reported 32 days.

Data in the literature suggest that the maximum recommended waiting time for repair of AAA larger than 8cm (diameter) and for revascularization of PAD with ischemic pain at rest is seven days; 14 days for repair of AAA with a diameter between 6 and 8cm; and 28 days for repair of AAA measuring less than 6cm in diameter.^(11)^ These guidelines were followed when scheduling the procedures during this project.

Regarding costs of the PPP, we analyzed the amounts spent with endovascular materials and hospital costs separately, because the amounts paid by the SUS to public hospitals that perform these surgeries are set per procedure and are divided into two items: endovascular material and hospital stay, including medical fees.

In regards to endovascular material the amounts spent during the project were higher than those paid by the SUS for the femoropopliteal and aneurysm groups. The determination of the SUS to reimburse a fixed value for supplies is based on an incorrect assumption that a limited number of equipment is used. This is not in line with reality, since it is not always possible to predetermine the number of supplies to be used^(12)^ and certainly this figure is greater than that expected by the SUS. This fixed value determines that only one stent may be used for each procedure. In practice, in order to obtain better clinical results we may use more than one stent per patient.

Evaluating hospital costs, we used the SUS table, and verified the following amounts paid to health facilities: R$ 1,541.28 to repair endovascular aneurysms, R$ 999.00 for lower limb angioplasties, and R$ 1,541.27 for iliac artery angioplasties. These values are fixed regardless of the surgical outcome or eventual complications. The intrahospital costs in the project were 884% more expensive for femoropopliteal angioplasties, 400% more expensive for iliac angioplasties, and 600% more expensive for aneurysm repairs (Table 2).

The intrahospital costs verified in our project relate to a hospital stay of only 1.1 days, in average. This is the most critical and expensive period in care of these patients. This value included the surgery and the immediate postoperative period at the ICU, but did not include medical fees and the complete admission period. This short period has already proven to be much more expensive than that paid by the SUS and demonstrates that values paid by the public health system are outdated.

Despite the fact that originally PPPs were established to deliver care, they can and must be complemented with scientific production. In addition to providing medical care and resolution to clinical problems of the patients involved, the PPPs should link scientific production to healthcare. In the case of this partnership, patient’s care was delivered according to a research protocol to assess a novel intravascular contrast medium (CO_2_) for endovascular procedures. The research protocol had been approved by the hospital Research Ethics Committee. The protocol of this PPP provided data showing that the use of CO_2_ may help the SUS reduce the cost of treatment for PAD patients.^(7)^

PPPs should be organized to allow the development of science and technology in healthcare combined with assistance, and may provide scientific production, quality and efficiency of care for public patients..

## CONCLUSION

Public-private partnerships are an effective alternative to speed up assistance and to improve the prognosis of patients suffering from vascular diseases, furthermore, those can minimize, at least partially, the structural deficit of the SUS. Private hospitals, duly equipped for this type of therapy, could absorb part of the public demand. The development of healthcare research may be included in these partnerships. The flows and costs described herein may contribute to establish future public-private partnerships in vascular surgery.
