# Supplementary material for: Public private partnership in vascular surgery
Source: Einstein (Sao Paulo). 2014 Jul-Sep;12(3):342–6. doi: 10.1590/S1679-45082014GS3029 (PMC4872947; doi:10.1590/S1679-45082014GS3029)
Supplement: Translation

# Translation: Parceria público-privada em cirurgia vascular

**Authors:** Cynthia de Almeida Mendes, Alexandre de Arruda Martins, Marcelo Passos Teivelis, Sérgio Kuzniec, Nelson Wolosker

**Affiliations:** 1 Hospital Israelita Albert Einstein, São Paulo, SP, Brasil.

**Keywords:** Procedimentos cirúrgicos vasculares/economia, Gestão em saúde, Sistema Único de Saúde, Angioplastia/economia, Aneurisma aórtico

## Abstract

**Objetivo:**

Descrever e analisar os resultados de parceria público-privada entre o Ministério da Saúde e um hospital privado em projeto de assistência e pesquisa científica na área de cirurgia endovascular. Métodos: Foram analisados fluxos, custos e resultados clínicos dos pacientes atendidos numa parceria público-privada entre abril de 2012 e julho de 2013. Todos os pacientes foram operados, ficaram pelo menos um dia na unidade de terapia intensiva do hospital privado e participaram de um protocolo de pesquisa para comparação entre dois contrastes endovenosos para cirurgia endovascular (contraste iodado e dióxido de carbono).

**Resultados:**

: Foram realizados 62 procedimentos endovasculares em 57 pacientes provenientes do sistema público. Os gastos hospitalares e com material endovascular mostraram-se significativamente maiores em relação ao que é pago pelo Sistema Único de Saúde (SUS) em dois dos três grupos de doenças estudados. Entre os pacientes ambulatoriais, o intervalo médio entre a consulta e a cirurgia foi de 15 dias e, nos internados, 7 dias. Todos os procedimentos foram bem sucedidos, sem conversão para cirurgia aberta. O novo contraste estudado, o dióxido de carbono, mostrou-se eficaz e mais barato.

**Conclusão:**

O tempo de espera dos pacientes entre indicação cirúrgica e sua realização foi significativamente reduzido. Parcerias público-privadas podem trazer agilidade no atendimento dos pacientes do SUS, permitindo também geração de conhecimento científico.

## INTRODUÇÃO

Doenças arteriais são enfermidades graves e que acometem com maior frequência indivíduos da sexta a oitava década de vida.^(1)^ A melhora nos serviços públicos de saúde propiciou um aumento na expectativa de vida da população;^(2)^ consequentemente as doenças arteriais estão cada vez mais prevalentes e geram grande demanda no Sistema Único de Saúde (SUS).

O tratamento dessas doenças é complexo, necessitando procedimentos cirúrgicos especializados realizados em centros terciários de atenção à saúde. Para a realização desses procedimentos, são necessários equipamentos sofisticados, equipes altamente preparadas e unidade de terapia intensiva (UTI) equipada para o pós-operatório.

Apesar dos grandes avanços do SUS, muitos pacientes enfrentam dificuldades para o acesso a esse tipo de tratamento, especialmente por limitações no número de vagas e de acesso a hospitais de alta complexidade.

Essa grande demanda de pacientes no sistema público e a capacidade de recursos limitada acarretam pior prognóstico para os pacientes. Silvany Neto et al. demonstraram que, em pacientes com doença arterial obstrutiva periférica (DAOP), o atraso no atendimento e na realização de exames gera um aumento no índice de amputações.^(3)^ O atraso de tratamento de pacientes internados também piora o desfecho e a mortalidade.^(4)^ Turnbull et al. e Oram et al. verificaram que o atraso no atendimento de pacientes portadores de aneurisma de aorta abdominal (AAA) pode aumentar o índice de ruptura e mortalidade.^(5, 6)^

Com o objetivo de melhorar o atendimento e o prognóstico desses doentes, a deficiência estrutural do SUS poderia ser parcialmente compensada por parcerias entre governo e hospitais privados, ou seja, parcerias público-privadas (PPP), nas quais hospitais privados, devidamente equipados e com toda estrutura necessária para esse tipo de atendimento, poderiam absorver parte da demanda pública.

Em 2009, o Hospital Israelita Albert Einstein (HIAE) estabeleceu uma PPP com o Ministério da Saúde por meio do Programa de Apoio ao Desenvolvimento Institucional do SUS (PROADI-SUS) para a realização de procedimentos endovasculares em pacientes do SUS portadores de oclusões arteriais fêmoro-poplíteas e aorto-ilíacas, além de aneurismas de aorta abdominal.

A finalidade dessa PPP era o tratamento de pacientes portadores de DAOP fêmoro-poplítea e aorto-ilíaca, e de AAA, disponibilizando a estrutura do HIAE para a realização dos procedimentos cirúrgicos e a recuperação pós-operatória, com base em uma pesquisa em saúde para avaliação de um novo contraste intra-arterial, o dióxido de carbono (CO_2_), para a realização desses procedimentos endovasculares.

## OBJETIVO

O objetivo do presente artigo foi descrever e analisar os resultados de parceria público-privada pioneira entre o Ministério da Saúde e um hospital privado em projeto de assistência e pesquisa científica na área de cirurgia endovascular, que pode servir como base para o planejamento de futuras parcerias público-privadas.

## MÉTODOS

Entre abril de 2012 e julho de 2013, 48 pacientes portadores de DAOP fêmoro-poplítea ou aorto-ilíaca, e 9 pacientes portadores de AAA foram tratados no HIAE por essa PPP, sendo submetidos a 62 procedimentos endovasculares.

Os pacientes tratados eram participantes de um projeto de pesquisa em saúde autorizado pelo Comitê de Ética em Pesquisa (CAAE: 00794012.5.0000.0071) do hospital. Todos tinham preenchido o Termo de Consentimento Livre e Esclarecido (TCLE), um dos critérios para inclusão no projeto.

Esses pacientes, na maioria, eram provenientes de Ambulatórios Médicos de Especialidades (AMES) das regiões do Capão Redondo, do Jardim São Luís e do Jardim Ibirapuera, na zona sul da cidade de São Paulo (SP), área de atendimento do Hospital Municipal M’boi Mirim − Dr. Moysés Deutsch (HMMD), que participou da PPP em parceria com o HIAE. Pacientes internados em outros hospitais da região que estavam aguardando procedimentos endovasculares também foram incluídos no projeto.

Previamente ao início do projeto, foi realizada uma ampla divulgação e orientação sobre o mesmo por meio dos gerentes médicos das unidades de saúde da região e, individualmente, com os médicos especialistas das unidades, para que se estabelecesse um fluxo de encaminhamento dos casos elegíveis.

Pacientes examinados nas unidades de saúde durante atendimento de rotina com cirurgião vascular, que fossem portadores dessas patologias e com indicação de correção cirúrgica foram encaminhados para ambulatório específico de triagem para o projeto, localizado nas dependências do HMMD. Os exames pré-operatórios foram solicitados por meio do ambulatório nas dependências do HMMD ou na Unidade Básica de Saúde (UBS), ambos pelo SUS. Angiotomografia pré-operatória dos pacientes foi realizada no Serviço de Radiologia do HMMD.

Médicos participantes do projeto, que atuavam tanto no HMMD quanto no HIAE, realizaram os atendimentos nos ambulatórios de triagem para o projeto.

Todos os casos triados foram discutidos pela equipe responsável pelo projeto no HIAE em reuniões semanais, durante as quais foi analisado se os critérios de inclusão eram contemplados. Pacientes encaminhados ao ambulatório do projeto, mas que não se enquadraram nos critérios de inclusão do estudo, foram reencaminhados ao serviço de origem para seguirem o fluxo habitual do SUS.

Uma vez incluído no projeto, o paciente teve sua cirurgia agendada no HIAE. A internação aconteceu no dia do procedimento e os pacientes foram encaminhados diretamente ao centro cirúrgico, na sala endovascular, onde foram realizados todos os procedimentos. A sala endovascular é totalmente equipada para a realização desses procedimentos, com equipamentos de fluoroscopia e anestesia. A qualidade dos equipamentos permitiu a realização segura dos procedimentos cirúrgicos e do estudo em relação ao contraste intravascular utilizado, comparando o novo contraste (CO_2_) ao contraste iodado convencional.^(7)^

Após o procedimento, o pacientes foram sistematicamente enviados à UTI do HIAE, onde permaneceram até o dia seguinte ao procedimento, quando, então, se não houvesse contraindicação clínica, foram transferidos ao HMMD, para outro hospital de origem ou até mesmo liberados para suas residências.

Os custos intra-hospitalares foram previamente estimados, pois todos os pacientes, independentemente do *status* clinico após o procedimento, foram encaminhados para permanência por 24 horas na UTI do HIAE.

Todo o material endovascular (cateteres, fios-guia, stents, balões e endopróteses) utilizado nos procedimentos foi quantificado para posterior análise de custos, bem como todo o contraste utilizado.

A transferência do paciente para o hospital de origem foi realizada por ambulância UTI vinculada ao projeto.

Após a alta hospitalar, os pacientes tratados pelo projeto permaneciam em seguimento pós-operatório nos serviços de origem e no ambulatório do projeto no HMMD.

A coleta de dados foi realizada em tempo real, com atualização semanal de planilha com todos os dados médicos, custos e evolução médica dos pacientes.

Todos os custos, em relação aos materiais endovasculares e de internação, foram registrados para posterior análise e comparação.

Foram analisadas as características demográficas da população atendida, os fluxos de encaminhamento de pacientes, o tempo de espera para a realização dos procedimentos, os custos hospitalares e aqueles com material endovascular.

## RESULTADOS

Foram realizados 62 procedimentos de abril de 2012 a agosto de 2013 em 57 pacientes provenientes do SUS, sendo 44 angioplastias fêmoro-poplíteas, 10 correções de AAA infrarrenal e 8 angioplastias aorto-ilíacas.

Desses pacientes, 44 eram provenientes das UBS da região do HMMD e 14 eram pacientes internados em outros hospitais de São Paulo, aguardando realização de procedimento endovascular.

A média de tempo entre o primeiro atendimento no ambulatório de triagem e o procedimento cirúrgico foi de 15 dias, variando de 5 a 25 dias. Nesse período, foram realizados todos os exames e avaliações pré-operatórias. No caso de pacientes internados, o tempo máximo de espera por procedimento foi de 7 dias.

Foram incluídos no projeto 37 pacientes do sexo masculino e 20 do feminino. A média de idade foi de 63,56 anos (variando de 37 a 82 anos). Dentre esses pacientes, 46 (80,7%) eram hipertensos, 26 (45,6%) diabéticos e 3 (5,2%) portavam insuficiência cardíaca congestiva. Hipercolesterolemia estava presente em 25 (43,8%), insuficiência coronariana em 8 (14%) e antecedente de tabagismo em 37 (64,9%).

Lesões tróficas associadas à dor isquêmica de repouso estiveram presentes em 42 pacientes com DAOP (37 com doença fêmoro-poplítea e 5 com acometimento aorto-ilíaco). Claudicação intermitente limitante esteve presente em três pacientes no grupo fêmoro-poplíteo e dois no aorto-ilíaco. Dor isquêmica de repouso sem lesão trófica esteve presente em um paciente no grupo aorto-ilíaco.

Apenas quatro pacientes necessitaram permanecer no HIAE por mais de 1 dia em UTI (um por mais 2 dias, e três por mais 1 dia). A duração média das internações no HIAE foi de 1,1 dia.

A mortalidade em 30 dias no grupo estudado foi de 4 casos (12%), todos eram pacientes portadores de DAOP fêmoro-poplítea que evoluíram com complicações cardíacas no pós-operatório.

Os gastos hospitalares por tipo de procedimento são apresentados na tabela 1.

**Tabela 1:** Gastos hospitalares por tipo de procedimento

Procedimento	Quantidade	Valor mínimo hospitalar (R$)	Valor máximo hospitalar (R$)	Valor médio hospitalar (R$)
AFP	44	4.780,44	34.333,42	8.834,36
AAO	8	2.769,46	10.737,0	6.218,00
CEAAAIR	10	6.207,27	15.400,89	9.258,78

AFP: angioplastias fêmoro-poplíteas; AAO: angioplastias aorto-ilíacas; CEAAAIR: correção endovascular de aneurisma de aorta abdominal infrarrenal.

Os maiores gastos ocorreram no grupo em que foi realizado o tratamento de aneurisma de aorta.

Os gastos com material endovascular por procedimento são apresentados na tabela 2.

**Tabela 2:** Gastos com material endovascular por procedimento

Procedimento	Quantidade	Valor mínimo material (R$)	Valor máximo material (R$)	Valor médio material (R$)
AFP	44	1.664,64	23.452,00	7.413,25
AAO	8	1.782,00	8.052,00	4.672,75
CEAAAIR	10	17.372,00	41.816,67	31.727,73

AFP: angioplastias fêmoro-poplíteas; AAO: angioplastias aorto-ilíacas; CEAAIR: correção endovascular de aneurisma de aorta abdominal infrarrenal.

Os gastos com material endovascular nos AAA foram quatro vezes mais caros que com angioplastias de membros inferiores e sete vezes mais caros que angioplastias ilíacas.

Os custos hospitalares e do material endovascular foram comparados com os valores de reembolso do SUS e são apresentados na tabela 3.

**Tabela 3:** Custos hospitalares e com material endovascular no Hospital Israelita Albert Einstein (HIAE) e valores de reembolso Sistema Único de Saúde (SUS)

Grupo	Tipo de gasto	n	Gasto HIAE (R$)	Reembolso SUS (R$)	Valor de p
			Média (DP)			
Aneurismas	Hospitalar	10	9.258,80 (3183,20)	1.541,30	<0,001
	Material	10	31.727,70 (7754,70)	25.455,00	0,031
Aorto-ilíaco	Hospitalar	8	6.218,00 (2.223,60)	1.541,30	0,001
	Material	8	4.672,70 (1.998,30)	5.340,00	0,376
Fêmoro-poplíteo	Hospitalar	44	8.834,40 (5.040,80)	999,00	<0,001
	Material	44	7.413,20 (4.444,00)	4.965,00	0,001

DP: desvio padrão.

Todas as despesas no HIAE mostraram-se significativamente maiores em relação ao reembolso SUS, com exceção do custo de material no grupo aortoilíaco.

## DISCUSSÃO

A DAOP sintomática e os AAA com mais de 5cm são doenças cada vez mais frequentes, que necessitam de tratamento cirúrgico. O número de pacientes aguardando cirurgia em nosso país é maior que o número de vagas disponibilizado pelo SUS, e o atraso no tratamento pode aumentar o índice de amputações e óbitos.^(3, 6)^

O fluxo estabelecido para o encaminhamento de pacientes neste projeto de PPP permitiu o tratamento de 57 doentes que aguardavam cirurgia na região sul da cidade de São Paulo. A integração de informação entre as UBS e o ambulatório do projeto foi fundamental para permitir que esses pacientes tivessem acesso ágil ao tratamento pelo qual aguardavam.

No serviço público, em razão da dificuldade de acesso desses pacientes a serviços em que pode ser realizado o tratamento que precisam, muitas vezes os pacientes são atendidos já com a doença avançada, e a demora para realização efetiva do tratamento piora ainda mais o prognóstico. Nunes et al. mostraram que 96,2% dos pacientes com DAOP internados em um serviço terciário já se apresentavam com lesões tróficas; em 62,3% as lesões eram tão extensas que não foi realizada nenhuma tentativa de revascularização antes da amputação.^(8)^

Um dos principais entraves na universalidade de atendimento no SUS é o acesso a um médico especialista, como um cirurgião vascular. Uma parceria com sistemas de saúde privados em PPP pode, como visto neste grupo de pacientes, minimizar esse problema.^(9)^

Os pacientes tratados por meio deste projeto eram oriundos de hospitais e UBS do SUS, constituindo uma amostra real da população atendida pelo sistema público e dos gastos públicos com esse tipo de doença. Esses pacientes geralmente são doentes graves, com muitas comorbidades associadas e com risco cirúrgico alto. Nesse projeto, a mortalidade foi de 12% − todos pacientes do grupo fêmoro-poplíteo, e esteve dentro do esperado pela literatura.^(10)^

Os procedimentos endovasculares realizados pelo nosso grupo, neste projeto, tiveram sucesso técnico de 100%, com todos as cirurgias sendo finalizadas com sucesso, sem necessidade de conversão para cirurgia aberta. O índice de salvamento de membros entre os pacientes com DAOP foi de 92%, com apenas quatro amputações e nenhuma complicação cirúrgica no grupo dos AAA.

O tempo de espera dos pacientes para a realização das cirurgias no projeto foi aquele necessário para a realização dos exames complementares pré-operatórios. Todo o pré-operatório foi realizado pelo SUS por meio do ambulatório do projeto no HMMD e consideramos que a espera nessa PPP (em média de 15 dias) foi muito curta, pois, apesar da literatura carecer de artigos recentes em relação a tempo de espera para cirurgias arteriais, estudo brasileiro anterior relatou espera média de 16,3 dias apenas para a realização de arteriografia pré-operatória (que, neste projeto, foi substituída pela angiotomografia) e de 32 dias para a realização de tentativa de revascularização para pacientes internados.^(8)^ Não existem estudos brasileiros que descrevam o tempo de espera de pacientes ambulatoriais para a realização desses procedimentos, mas, em serviços públicos da cidade de São Paulo (onde essa população específica seria atendida), essa espera pode ser ainda maior que os 32 dias previamente relatados.

Dados em literatura sugerem que o tempo máximo recomendado de espera são 7 dias para correção de AAA com mais de 8cm de diâmetro e para revascularização de DAOP com dor isquêmica de repouso; 14 dias para correção de AAA com 6 a 8cm de diâmetro; e 28 dias para correção de AAA com menos de 6cm de diâmetro.^(11)^ Essas diretrizes foram seguidas para os agendamentos deste projeto.

Em relação aos custos da PPP, fizemos a análise dos valores gastos com material endovascular e intra-hospitalar separadamente, pois os valores pagos pelo SUS aos hospitais públicos que realizam essas cirurgias são fixos por procedimento e divididos em dois itens: material endovascular e internação hospitalar, incluindo honorários médicos.

Em relação ao material endovascular, os valores gastos no projeto foram maiores que aqueles pagos pelo SUS no grupo fêmoro-poplíteo e aneurismas. Quando o SUS estabelece os valores fixos de reembolso de material, ele se baseia numa determinação equivocada do uso de um número limitado de equipamentos, o que não condiz com a realidade, visto que esse número não é totalmente previsível ^(12)^ e, com certeza, é maior que o previsto pelo SUS. Esse valor fixo limita, por exemplo, o uso de apenas um stent por procedimento. Na prática, podemos utilizar mais de um stent por caso, para obter melhores resultados clínicos.

Sobre os custos hospitalares, utilizando-se como base a mesma tabela do SUS, observamos que a remuneração repassada aos serviços de saúde é R$ 1.541,28 por correção de aneurisma endovascular, R$ 999,00 por angioplastia de membros inferiores, e R$ 1.541,27 por angioplastia de artéria ilíaca. Esses também são valores fixos, que independem do desfecho dos procedimentos ou da ocorrência de complicações. Os valores intra-hospitalares gastos no projeto mostraram-se 884% mais caros com relação às angioplastias fêmoro-poplíteas; 400% mais caros nas angioplastias ilíacas; e 600% mais caros em relação aos aneurismas (Tabela 2).

Os valores aferidos em nosso projeto, com relação aos custos intra-hospitalares, decorreram de uma internação de apenas 1,1 dia em média, sendo este o período mais crítico e caro do tratamento desses doentes. Esse valor incluiu a realização da cirurgia e o pós-operatório imediato em UTI, mas não incluiu a internação total do doente nem honorários médicos. Esse curto período já se mostrou muito mais caro do que o valor pago pelo SUS e demonstra a defasagem dos valores pagos pelo sistema público aos serviços de saúde.

PPPs, apesar de originalmente assistenciais, podem e devem ser complementadas com produção científica, proporcionando, além do atendimento médico e da resolução do problema clínico dos pacientes envolvidos, uma produção cientifica agregada aos atendimentos. Nessa PPP, o atendimento aos pacientes era realizado dentro de um protocolo de pesquisa, aprovado pela comissão de ética do hospital, sobre um novo contraste intravascular (CO_2_) para realização de cirurgias endovasculares. Com os dados obtidos por esse protocolo, cuja realização foi possível pela existência da PPP, pudemos observar que o uso do CO_2_ pôde gerar uma economia ao SUS no tratamento dos pacientes com doença arterial periférica.^(7)^

As PPP devem ser organizadas de modo a permitirem o desenvolvimento de ciência e tecnologia na área da saúde, aliadas à assistência, podendo proporcionar produção científica, agilidade e qualidade de atendimento para os pacientes do SUS.

## CONCLUSÃO

Parcerias público-privadas são uma alternativa efetiva para agilizar o atendimento e o prognóstico de pacientes com doenças vasculares, de maneira a compensar, ao menos parcialmente, a deficiência estrutural do SUS. Hospitais privados e devidamente equipados para esse tipo de atendimento poderiam absorver parte da demanda pública. O desenvolvimento de pesquisa em saúde pode ser realizado em conjunto com essas parcerias. Os fluxos e custos aqui descritos podem auxiliar na elaboração de futuras parcerias público-privadas em cirurgia vascular.
